# Lymph node metastasis determined miRNAs in esophageal squamous cell carcinoma

**DOI:** 10.18632/aging.206122

**Published:** 2024-10-14

**Authors:** Feng Wei, Shufeng Bi, Mengmeng Li, Jia Yu

**Affiliations:** 1 Department of Critical Care Medicine, Affiliated Hospital of Chifeng University, Chifeng 024000, Inner Mongolia Autonomous Region, China; 2 Department of Chronic Disease, Chifeng Center for Disease Control and Prevention, Chifeng 024000, Inner Mongolia Autonomous Region, China

**Keywords:** esophageal squamous cell carcinoma, lymphatic metastasis, MicroRNAs, computer algorithm

## Abstract

Purpose: There is no golden noninvasive and effective technique to diagnose lymph node metastasis (LNM) for esophageal squamous cell carcinoma (ESCC) patients. Here, a classifier was proposed consisting of miRNAs to screen ESCC patients with LNM from the ones without LNM.

Methods: miRNA expression and clinical data files of 93 ESCC samples were downloaded from TCGA as the discovery set and 119 ESCC samples with similar dataset GSE43732 as the validation set. Differentially expressed miRNAs (DE-miRNAs) were analyzed between patients with LNM and without LNM. LASSO regression was performed for selecting the DE-miRNA pair to consist the classifier. To validate the accuracy and reliability of the classifier, the SVM and AdaBoost algorithms were applied. The CCK-8 and wound healing assay were used to evaluate the role of the miRNA in ESCC cells.

Result: There were 43 DE miRNAs between the LNM+ group and LNM- group. Among them, miR-224-5p, miR-99a-5p, miR-100-5p, miR-34c-5p, miR-503-5p, and miR-452-5p were identified by LASSO to establish the classifier. SVM and AdaBoost showed that the model could classify the ESCC patients with LNM from the ones without LNM precisely and reliably in 2 data sets. miR-224-5p in the classifier as the top contributor to discriminate the two groups of patients based on AdaBoost, promoted ESCC cell proliferation and migration *in vitro*.

Conclusion: The classifier based on these 6 miRNAs could classify the ESCC patients with LNM from the ones without LNM successfully.

## INTRODUCTION

### Background

Esophageal cancer (EC) is one of the most common digestive tract tumors, the mortality and morbidity stated as the 6th and 7th in the world, respectively [1]. Esophageal squamous cell carcinoma (ESCC) is the main subtype of EC, accounting for nearly 80% of EC patients and the 5-year survival rate of most ESCC patients present with advanced disease is among 5–20% [2]. There has been a change in the incidence rate over the past few decades. ESCC is not only frequently seen in developing countries, but has had a dramatic increase in the western world, just in the United States with 16,940 new cases and 15,690 deaths every year [3]. There is a clinical concern about ESCC that poor 5-year overall survival rates are associated with this malignancy despite the advance in management and treatment [4, 5]. Lymph node metastasis (LNM) is one of the clinical risk factors that contributed the most to poor prognosis [6, 7]. ESCC patients with LNM have more chance to progress to loco-regional and distant recurrence which leads to a worse prognosis [8, 9]. The overall 5-year survival rates of ESCC patients with LNM would drop from 70–92% to 18–47% after surgery [6, 10–12]. The clinical decision of esophagectomy is heavily based on the status of LNM [7, 13]. Meanwhile, knowledge of LNM status is essential to decide whether ESCC patients can be cured with endoscopic resection alone without the need of other therapies [7, 14]. Hence, accurate discrimination of the patients with LNM from those without LNM for determining clinical strategies and prognostic outcomes is crucial. Until now, a gold standard modality of the LNM diagnosis strategy barely exists. Approximately 40% of patients are unable to detect the micro-metastases by the non-invasive conventional imaging techniques, which leads to a poor prognosis [15, 16]. There is a need to study the molecular biomarkers for detecting LNM status.

### Rationale and knowledge gap

MicroRNAs (miRNAs), showing an emerging direction of untraditional diagnosis, are giving innovative insights into the detection of cancer. Many efforts have been made to evaluate miRNAs to detect tumors in their early stage, such as for non-small-cell lung cancer, colorectal cancer, and ESCC [17–20]. Meanwhile, some models were established based on the miRNAs to predict the probability of LNM for cancer patients [21–23]. Until now, no such model had been established to detect the probability of LNM for ESCC patients.

The least absolute shrinkage and selection operator (LASSO) is one type of regression that selects a reduced group of the covariates to establish the classification model [24]. Support vector machines (SVMs) are one of the supervised learning models which analyze data for classification and ensure accuracy [25]. Adaptive Boosting (AdaBoost), is an algorithm to evaluate performance as well as rank the importance of factors in the classifier [26]. The classifier constructed by LASSO and verified by SVM and AdaBoost is reliable as a statistical predictive model that accurately classifies the probability of clinical events [27, 28].

### Objective

In the present study, we aimed to identify the miRNAs entered into a classifier that can be used to discriminate the ESCC patients with LNM from the patients without LNM by LASSO. Then the accuracy and reliability of the classifier were evaluated by SVM and AdaBoost. Furthermore, the factors in the model were ranked according to the contribution to the classification function by AdaBoost. The target genes of miRNA have been predicted by 3 prediction websites, and further pathway analyses that these target genes may be involved in were carried out by Gene Ontology (GO) and Kyoto Encyclopedia of Genes and Genomes (KEGG). Meanwhile, the function of over-expressed miRNA on proliferation and migration of TE-1 (ESCC cell) was explored.

## MATERIALS AND METHODS

### Collection and preparation of the data

93 ESCC tissue samples with the corresponding clinical information were downloaded from The Cancer Genome Atlas (TCGA) data portal (https://portal.gdc.cancer.gov/; IlluminaHiseq platform) as a discovery group to establish the classifier that was applied to discriminate the ESCC patients with LNM from the ones without LNM and GSE43732 including miRNA expression profiles of 119 ESCC frozen tumor tissue samples with clinical information from National Center for Biotechnology Information (NCBI) Gene Expression Omnibus (GEO) based on Agilent-038166cbc_human_ miR18.0 platform (https://www.ncbi.nlm.nih.gov/geo/query/acc.cgi?acc=GSE43732) as a validation set to verify the classifier.

miRNA-expression data of TCGA set and GSE43732 set were normalized and log_2_-transformed for the high and low expression of miRNAs, applying log-rank *p* < 0.05 as the cutoff.

### Screening for DE-miRNAs

From the TCGA database, 38 ESCC patients with LNM formed the experimental set and 55 ESCC patients without node metastasis formed the control set. After the normalization of miRNA expression data, differential expression analysis was performed between the 2 groups by the “limma” package in R 4.0.3. The miRNAs with *p* < 0.05, |log_2_FC|>1, and false discovery rate (FDR) *q* < 0.1 were identified as significant DE-miRNAs.

### Construction of the classifier

The LASSO regression was applied to identify DE-miRNAs for establishing the classifier within the TCGA set by glmnet package of R. Discrimination capability of the classifier was evaluated by area under the ROC curve (AUROC).

### Verification of the classifier

SVM and AdaBoost were to verify the classifier by R package of the e1071 and adabag. The efficacy and accuracy of the classifier were evaluated by kappa, accuracy, sensitivity, specificity, positive predictive value (PPV), and negative predictive value (NPV) in the TCGA database (*n* = 93) and GSE43732 dataset (*n* = 119). Furthermore, all factors in the classifier were given the sort order by Adaboost.

### Function analysis

Target genes of the ranked miRNA were predicted using miRDB (http://mirdb.org/), tarbase (https://ngdc.cncb.ac.cn/databasecommons/), and TargetScan (https://www.targetscan.org/) and overlapped target genes were obtained by taking the intersection through the website of Venn (http://bioinformatics.psb.ugent.be/webtools/Venn/). For the predicted targeted genes, gene ontology (GO) function and Kyoto Encyclopedia of Genes and Genomes (KEGG) pathway enrichment analyses were performed by the data from the database for annotation, visualization, and integrated discovery (DAVID) and the “tidy” package in R 4.1.3.

### Cell culture and transfection

The human esophageal cancer cell line TE-1 was purchased from the Cancer Institute (Chinese Academy of Medical Sciences, Beijing, China). TE-1 cells were cultured in a DMEM medium containing 10% fetal bovine serum, 37°C, and a 5% CO_2_ incubator in the closed culture. TE-1 cells were transfected with miR-224-5p mimics and negative controls (NCs) (GenePharma, Suzhou, China). The sequence of sense miR was 5′-UCAAGUCACUAGUGGUUCCGUUUAG-3′ and antisense was 5′-AAACGGAACCACUAGUGACUUGAUU-3′. GP-Transfected mate (GenePharma, Suzhou, China) as the cell transfection reagent was transfected with miRNA using the manufacturer’s instructions.

### Cell proliferation assay

TE-1 cells were seeded in the 6-well plate first, 24 h after transfection, and the cells were reseeded to a 96-well plate at a density of 6 × 10^3^ cells per well. A Cell Counting Kit-8 assay (CCK-8) was performed at 0 h, 24 h, 48 h, and 72 h, respectively. The absorbance of the 450 nm laser was measured after a 2 h incubation of cells with CCK-8. Each group had 3 repeats and the experiments were performed in triplicate.

### Wound healing assay

In 6-well plates, transfected cells were cultured (1 × 10^6^ cells/well). Before a scratch was made with the 20 µL pipette tip, cells were starved for 24 hours in a serum-free medium after it reached 90%. At 0 h, 12 h, and 24 h, photos were taken (Olympus, Tokyo, Japan) and the relative cell migration rate was calculated based on the filled wounded area.

### Statistical analysis

Data are expressed as the mean ± standard deviation (SD) from three independent replicates. The student’s *t*-test was used to compare the two groups of samples. The log-rank test was used to define the subgroups based on the expression of miRNAs. All statistical analyses were performed using R software (version 4.1.3) and pictured by GraphPad Prism version 5.0. *p* < 0.05 by 2 sides was regarded as statistically significant.

## RESULTS

### Identification of DE-miRNAs

**Figure 1:**
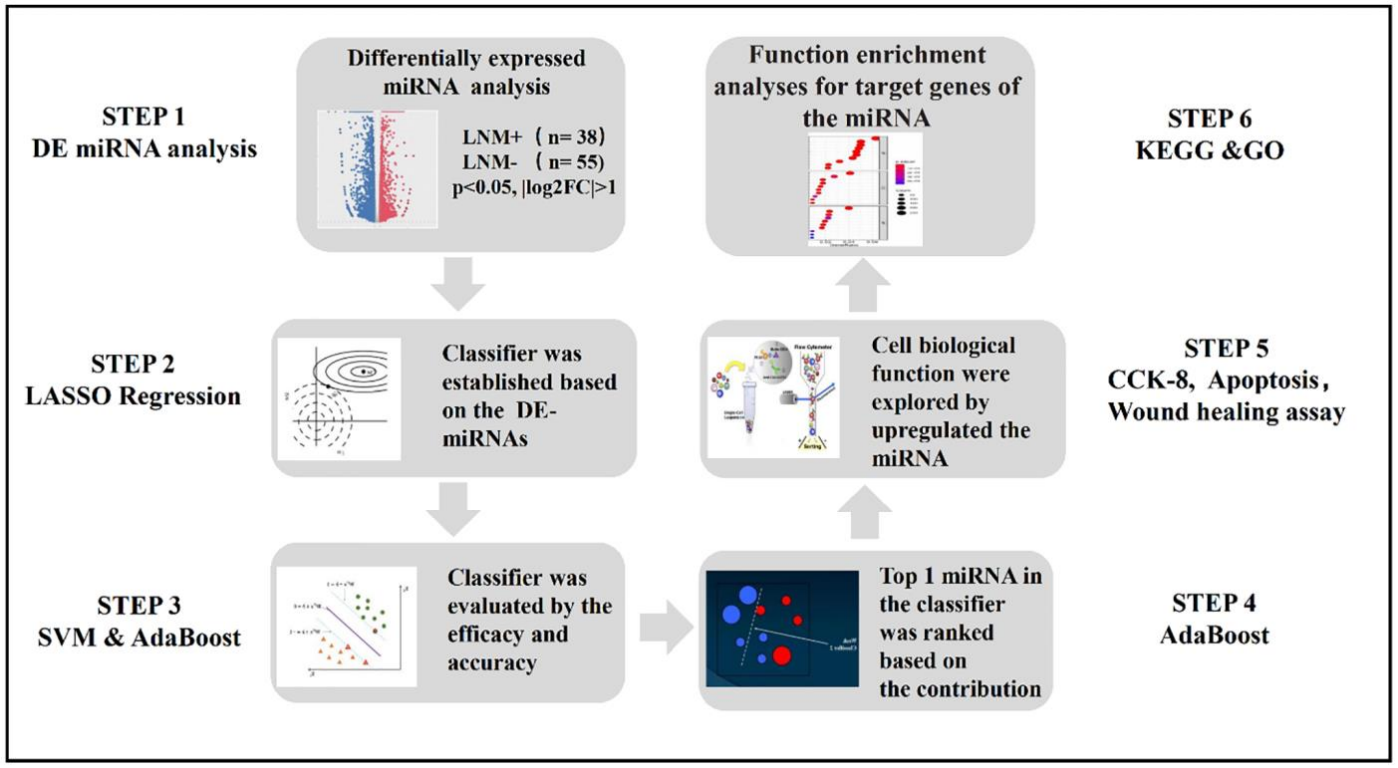
The flowchart of this study.

**Figure 2:**
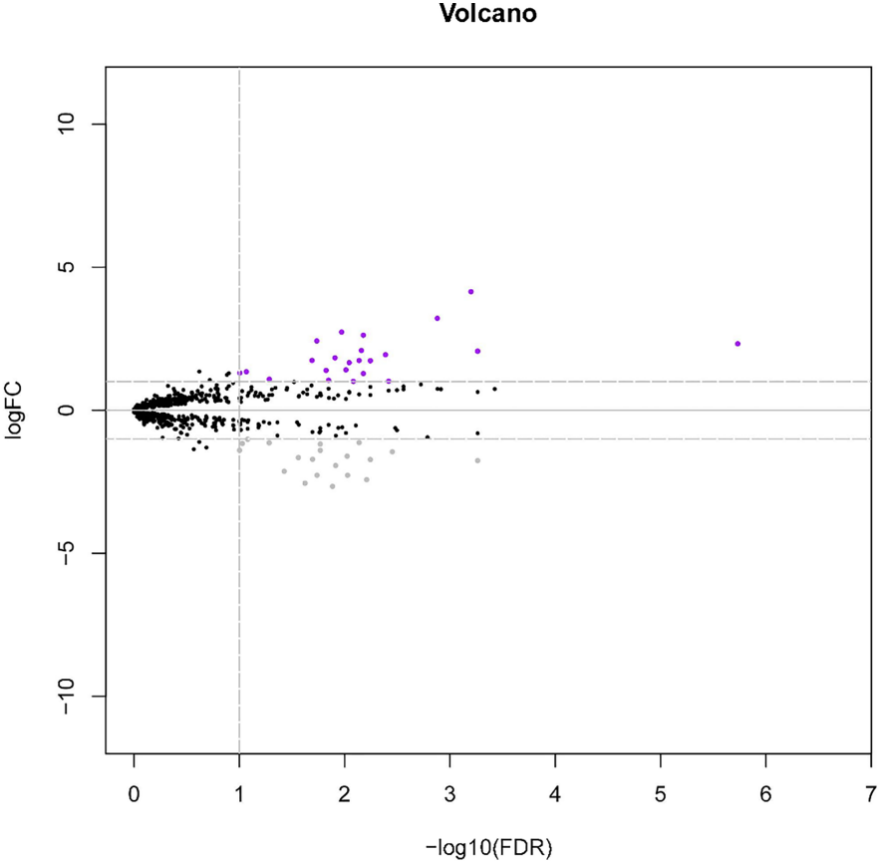
**The volcano plot of differentially expressed miRNAs (DE-miRNAs) between ESCC patients with LNM and without LNM from the TCGA dataset.** The purple dots and grey dots represent upregulated DEs and downregulated DEs with significance (adjusted *p*-value < 0.05 and |log2 (FC)| > 1), respectively. The black dots are those DEs without significance.

The overall study design was depicted in Figure 1. A total of 43 DE-miRNAs between the LNM + (*n* = 38) and LNM − (*n* = 55) samples were acquired, consisting of 20 down-regulated miRNAs and 23 up-regulated miRNAs in the patients with LNM compared to the ones without LNM. The volcano map indicated that the expression of these DE-miRNAs was significantly different between the 2 groups (Figure 2).

### Destruction of a classifier by LASSO

**Figure 3:**
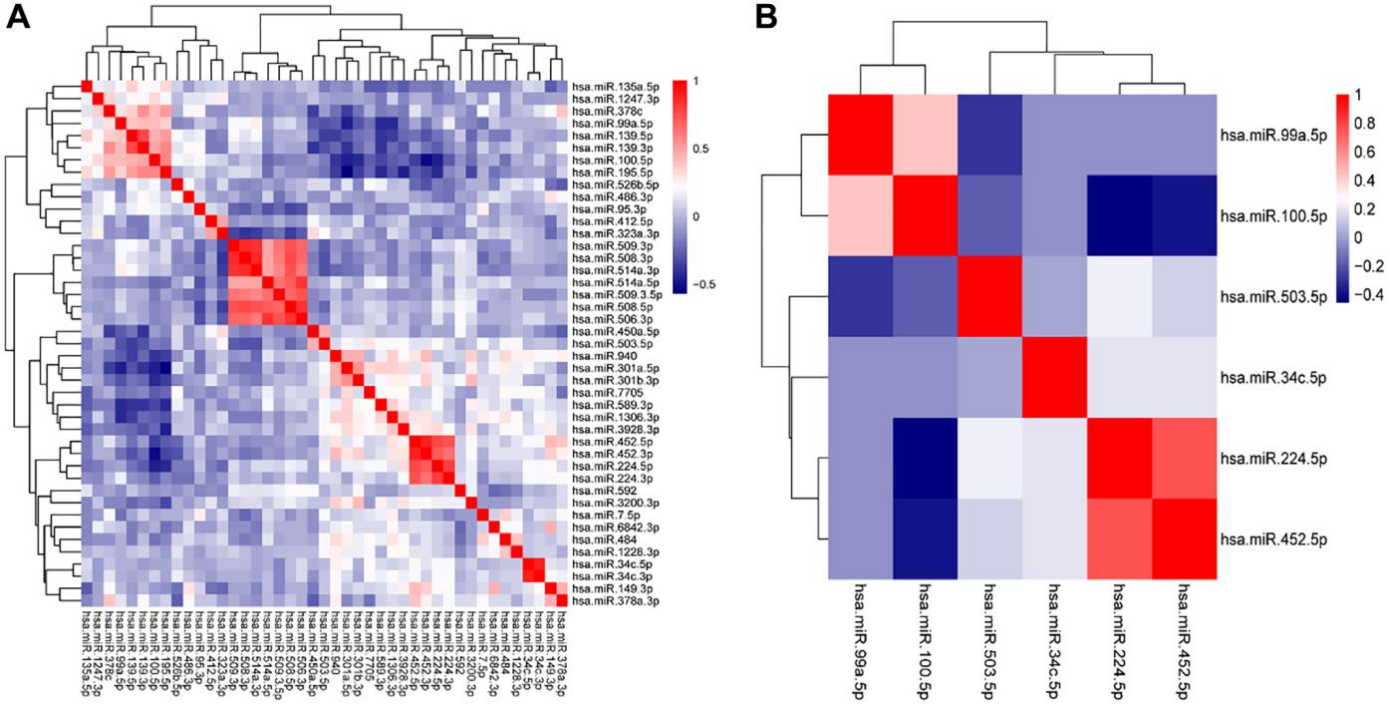
**Variable selection by LASSO.** (**A**) Hierarchical clustering shows the correlation matrix heatmap of 43 DE-miRNAs from the differential expression analysis. (**B**) Hierarchical clustering shows the correlation matrix heatmap of 6 DE-miRNAs which consisted of the classifier from LASSO regression analysis. Each cell represents the Pearson correlation between the row and column corresponding miRNAs. The legend shows the color change along with the change of correlation coefficient from 0.0 to 1.0.

**Figure 4:**
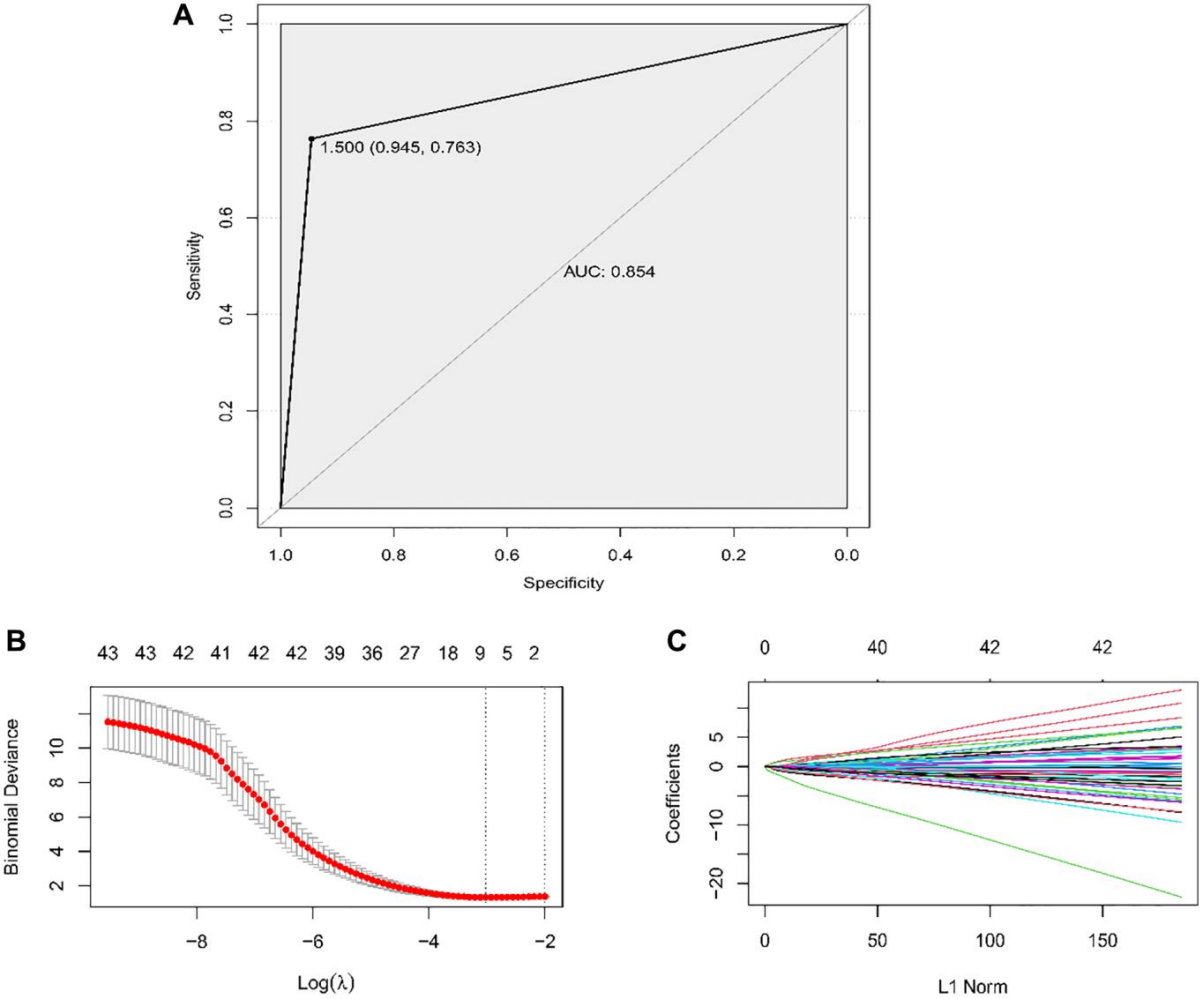
**Establishment of the classifier by LASSO.** (**A**) ROC curve to evaluate the discrimination ability of the classifier constructed by LASSO regression to different ESCC patients with LNM from the ones without LNM. (**B**) The vertical dashed lines were calculated at the best log (lambda) value and (**C**) LASSO coefficient values.

By the TCGA dataset, LASSO regression was applied to filter the optimal combination. With a min lambda of 0.048 obtained by performing 1000 cross-validations, 6 miRNAs (AUROC = 0.854) among 43 DE-miRNAs consisted the classification model which were miR-224-5p, miR-99a-5p, miR-100-5p, miR-34c-5p, miR-503-5p, and miR-452-5p (Figures 3A, 3B and 4A–4C).

### Verification of the classifier by SVM and Adaboost

**Figure 5:**
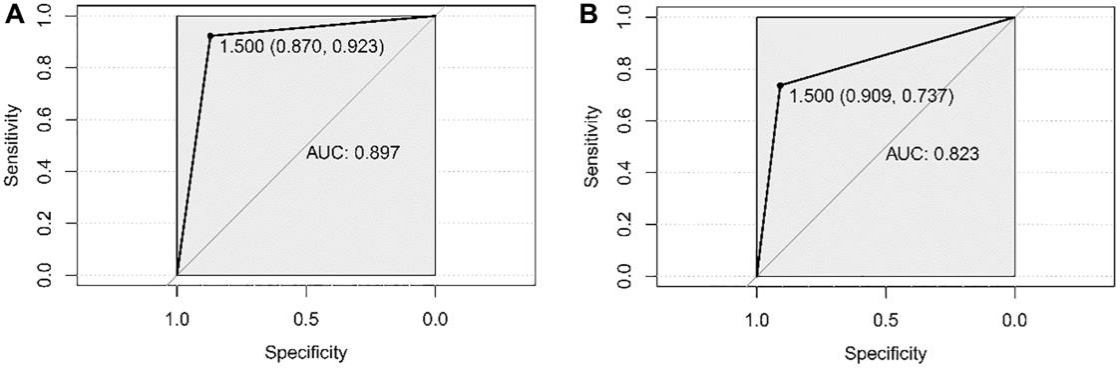
**Verification of the classifier by SVM.** (**A**) ROC curve to evaluate the discrimination ability of the classifier constructed by the SVM algorithm to different ESCC patients with LNM from the ones without LNM in the TCGA dataset. (**B**) ROC curve to evaluate the discrimination ability of the classifier constructed by the SVM algorithm to different ESCC patients with LNM from the ones without LNM in GSE43732.

The constructed classifier was verified by SVM and Adaboost on both the TCGA dataset (training set) and GSE43732 (validation set). Based on the SVM algorithm, the model could discriminate the patients with LNM from those without LNM in both datasets. This is evident by AUROC in the TCGA set of 0.823, and in the GSE43732 set of 0.897 (Figure 5A, 5B). As Table 1 shows, the classifier reliably distinguished the two groups as evident by: kappa (0.719); accuracy (0.838); sensitivity (0.909); specificity (0.736); PPV (0.833); and NPV (0.848).

**Table 1:** The validation of the classifier by SVM and Adaboost.

**Index**	**Discovery cohort**		**Validation cohort**	
	**SVM**	**AdaBoost**	**SVM**	**AdaBoost**
Kappa	0.719	0.906	0.796	0.904
Accuracy	0.838	0.896	0.899	0.963
Sensitivity	0.909	0.854	0.870	0.881
Specificity	0.736	0.836	0.923	0.815
Pos Pred Value	0.833	0.824	0.903	0.884
Neg Pred Value	0.848	0.878	0.895	0.854
*p*-Value (Acc > NIR)	<0.001	<0.001	<0.001	<0.001

Discovery cohort: TCGA dataset; Validation cohort: GSE43732 dataset.

From Adaboost, the classifier was with favorable accuracy (accuracy = 0.896, 0.963 in TCGA and GSE43732) and reliability (kappa = 0.906, 0.904 in 2 sets) as well, more details were shown in Table 1.

### Ranking the importance of factors in the classifier by Adaboost

**Figure 6:**
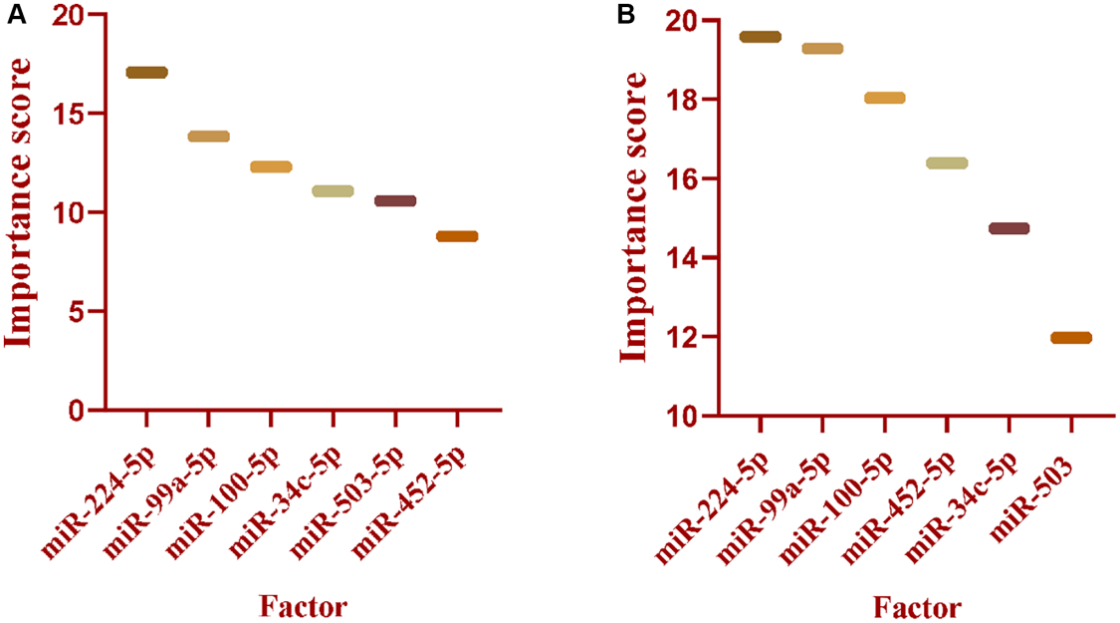
**Sorted the miRNAs in the classifier based on the contribution by Adaboost.** (**A**) The miRNAs in the classifier were ranked by Adaboost based on the contribution in TCGA dataset. (**B**) The miRNAs in the classifier were ranked by Adaboost based on the contribution in GSE43732.

The factors in the classifier were ranked by the AdaBoost algorithm on both the TCGA dataset and the GSE43732 set. In the TCGA set, miR-224-5p was the most significant one for discriminating the patients with LNM from the ones without LNM, followed by miR-99a-5p, miR-100-5p, miR-34c-5p, miR-503-5p, and miR-452-5p (Figure 6A). For the GSE43732 group, miR-224-5p contributes more to classify the patients into 2 groups than other miRNAs, miR-99a-5p, miR-100-5p, miR-452-5p, miR-34c-5p, and miR-503 (Figure 6B).

### Function analysis by KEGG and GO

**Figure 7:**
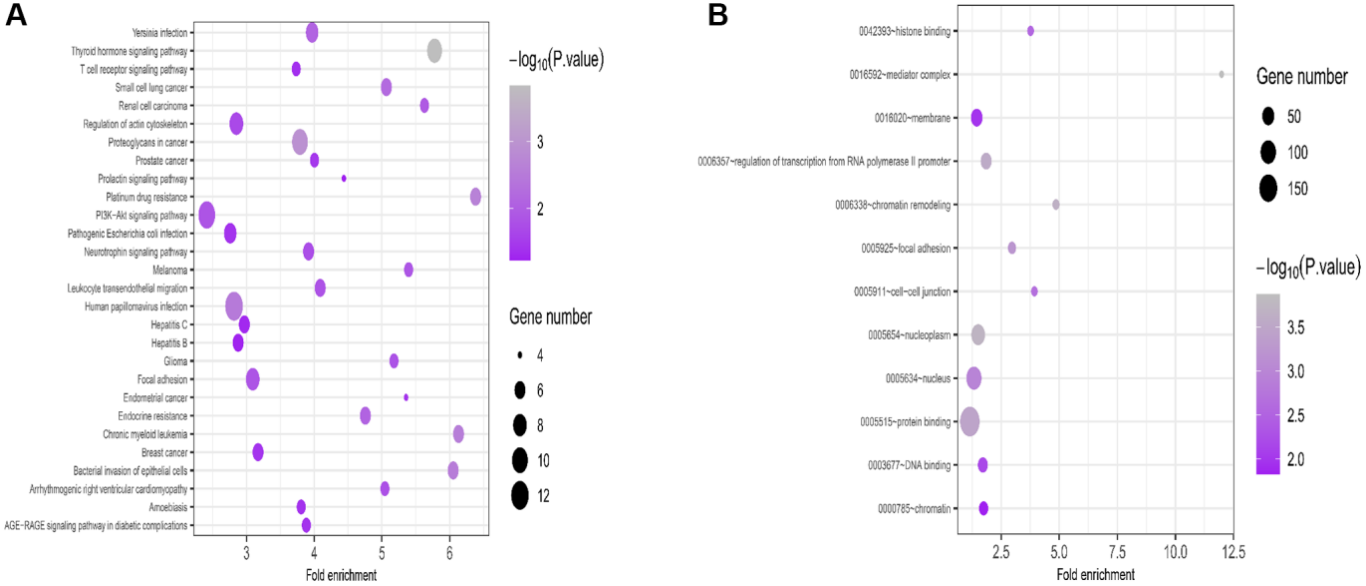
**GO and KEGG analysis for targeted genes of miR-224-5p.** (**A**) KEGG pathway analysis was performed for the candidate target genes. (**B**) Biological process enrichment analysis of candidate target genes.

KEGG pathway analysis revealed that the most enriched pathways for miR-224-5p target genes were the PI3K-Akt signaling pathway, human papillomavirus infection, and proteoglycans in cancer (Figure 7A). According to the GO analysis, they were engaged in protein binding, nucleus, and membrane formation (Figure 7B).

### Cell proliferation and migration assay

**Figure 8:**
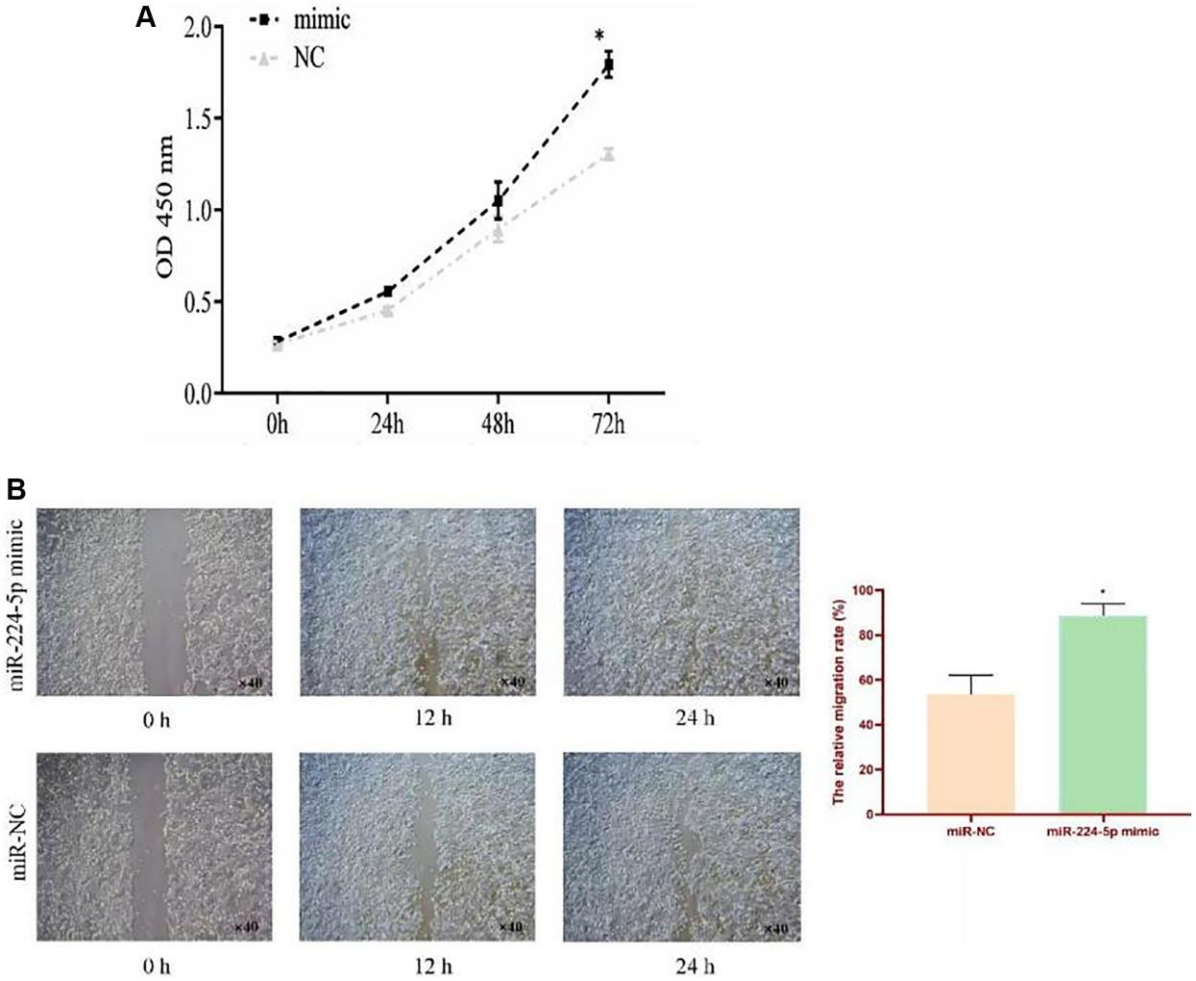
**Effect of miR-224-5p on proliferation and migration in cultured ESCC cell.** (**A**) CCK8 assays were performed at 0 h, 24 h, and 48 h after the transfection of TE-1 cells with miR-224-5p mimic and miR-NC. (**B**) Wound healing assay of the relative migration rate of TE-1 cells transfected with miR-224-5p mimic and miR-NC. ^*^p < 0.05.

Over-expressed miR-224-5p significantly promoted TE-1 cell proliferation compared to the NC group (Figure 8A). In addition, the wound healing assay showed that the miR-224-5p mimic group had an enhanced TE-1 cell migration rate (Figure 8B). Taken together, these results suggest that miR-224-5p may act as an oncogene to promote ESCC progression.

## DISCUSSION

### Key findings

In this study, we used 2 nationwide cohorts of ESCC patients to develop and validate a classifier consisting of 6-miRNA to discriminate the patients with LNM and without LNM. The present classifier successfully stratified patients into the group with LNM and without LNM in both the discovery cohort and the validation cohort as evidenced by values of C-index, AUROC, accuracy, kappa, sensitivity, specificity, PPV, and NPV. Furthermore, the largest contributor to the present classifier is miR-224-5p which might play a positive role in ESCC cell proliferation and migration.

### Limitations

In the present study, some limitations should be considered. Even though the external validation cohort was available, the patients were from United States rather than from other countries; the population in other countries are needed to validate the classifier. Moreover, the demographic factors, such as alcohol and smoking, which are well-known risk factors, were not analyzed in the present study through the classifier with favorable discrimination usage. Furthermore, miR-503-5p as one of the contributors was identified by LASSO in TCGA, however, in GSE43732, only miR-503 was available. Even though miR-503-5p belongs to the miR-503 cluster, there is a need to verify it in the other validation group. The samples and clinical information of ESCC patients from Affiliated Hospital of Chifeng University and Chifeng Oncology Hospital were collected to overcome these limitations.

### Comparison with similar researches

miRNAs have emerged as vital biomarkers due to their tumor and tissue specificity, their ability to resist RNase-mediated degradation (possibly due to their short length), and their intact expression in tissues as well as in bodily fluids (including blood samples). There was a study based on the miRNA signatures to build a classifier to detect LNM for T1b gastric cancer (GC) patients [29]. In that study, different miRNAs were analyzed between patients with LNM and without LNM, then the LASSO regression model was used to select miRNAs to establish the classifier. As the AUROC value reaching to 0.843, the classifier might have favorable discrimination usage based on the LASSO regression analysis. However, in that study, only the LASSO regression was applied to build and validate the model. In the present study, to ensure the accuracy and reliability of the classifier, the DE-miRNAs were analyzed between ESCC patients with LNM (+) and LNM (−) firstly, then the LASSO algorithm was applied to build the classifier by filtering among the 43 DE-miRNAs, followed by SVM and AdaBoost serving as the validation algorithms to verify the discrimination usage of the 6-miRNAs classifier. Finally, with all these algorithms, a precise and reliable classifier based on miR-224-5p, miR-100-5p, miR-99a-5p, miR-452-5p, miR-34c-5p, and miR-503 was established.

### Explanations of findings

The exertion-type of miR-224-5p mostly depends on the tissues or organs. For example, it acts as an oncogene in gastric cancer [30]; whereas it displays a tumor-suppressive role in prostate cancer and lung cancer [31, 32]. Moreover, miR-224-5p performed a positive role in radiotherapy and chemotherapy resistance in laryngeal carcinoma [33]. Zang et al. pointed out that up-regulated miR-224-5p was related to the higher probability of LNM in papillary thyroid carcinoma [34] and predicted the less favorable event-free survival (EFS) in ovarian cancer [35]. From the Adaboost, miR-224-5p is the top contributor to different ESCC patients with LNM from the ones without LNM. Besides that, it promoted ESCC cell proliferation, migration, and invasion *in vitro*. To explore its functional roles on ESCC, we predicted the target genes of miR-224-5p and analyzed the signaling pathways as well as possible biological processes which were involved in these target genes. KEGG and GO showed that they might be functionally related to the PI3K-Akt signaling pathway, human papillomavirus, and proteoglycans in cancer. Several studies verified that ESCC contributed to the abnormal activation of the PI3K-Akt signaling pathway [36–39]. The human papillomavirus (HPV) might relate to the incidence of ESCC, especially, infection by HPV16 and 18 which are associated with higher ESCC incidence risk compared to other HPV types. HPV16 infection promotes the invasion and migration of ESCC through mediating tumor-associated macrophages. The proteoglycans were associated with the promotion of ESCC progression. Nevertheless, cell functional research is needed to confirm the findings of our study.

miR-99a-5p was decreased, expressed in several tumors, such as ESCC, breast cancer, bladder cancer, etc. [40–42]. The down-regulation of miR-99a-5p was related to a poor prognosis for ESCC patients [40]. miR-452-5p expression was related to TNM staging, and patients who had high expression of it might have a poorer prognosis time in colorectal cancer [43]. miR-503 was illustrated as performing the restraining role in different tumor tissues, which was also related to the tumor size, stage, metastasis status, and prognosis [44]. miR-100-5p acted as an accelerator to cardiac hypertrophy, heart failure, and human articular chondrocytes through several pathways [45–47]. Several studies focused on cancer showed the function of miR-34c-5p as a tumor suppressor [48, 49]. Besides that, Kaiyou Fu, et al. illustrated that miR-34c-3p and miR-34c-5p might serve as biomarkers for detecting the LNM in endometrial cancer.

### Implications and actions needed

The classifier had good performance on discriminating the ESCC patients with LNM from the ones without LNM. The onco-role of hub miRNA (miRNA-224-5p) in the model was explored only by the *in vitro* experiments, the *in vivo* ones were not carried out. Further studies are urged to clarify underlying functions.

## CONCLUSION

The classifier consisting of these 6 miRNAs was established and validated; it can be applied to discriminate the ESCC patients with LNM from the ones without LNM with favorable accuracy and reliability. The classifier provided significant diagnostic value to existing diagnosis strategies and may assist in more individualized diagnosis for these patients.
